# Case Report: “Clipping” an Internal Carotid Artery Aneurysm With a Duplicated Middle Cerebral Artery and the Anterior Choroidal Artery Arising From the Dome

**DOI:** 10.3389/fneur.2022.845296

**Published:** 2022-03-03

**Authors:** Nozomi Otsuka, Hirohisa Yajima, Satoru Miyawaki, Satoshi Koizumi, Satoshi Kiyofuji, Hiroki Hongo, Yu Teranishi, Taichi Kin, Nobuhito Saito

**Affiliations:** ^1^Department of Neurosurgery, Faculty of Medicine, The University of Tokyo, Tokyo, Japan; ^2^Department of Medical Information Engineering, Graduate School of Medicine, The University of Tokyo, Tokyo, Japan

**Keywords:** duplicated middle cerebral artery, cerebral aneurysm, anterior choroidal artery, branch incorporated aneurysm, clipping, fusion three-dimensional computer graphics

## Abstract

**Background:**

A duplicated middle cerebral artery (DMCA) is an anatomical variant that includes duplication of the middle cerebral artery (MCA) and an anomalous vessel originating between the anterior choroidal artery (AChA) and the distal end of the internal carotid artery (ICA). Here, we present a case report of an ICA aneurysm with a DMCA and the AChA originating from the dome, which was successfully treated with clipping.

**Case Description:**

In a 64-year-old man, preoperative angiography revealed an unruptured right ICA aneurysm with a maximum diameter of 4.3 mm, and fusion three-dimensional computer graphics revealed that a DMCA and the AChA originated from the dome. The aneurysm enlarged; therefore, clipping was performed. The closure of the aneurysm while preserving the patency of the DMCA and AChA was identified using intraoperative microvascular Doppler ultrasonography and indocyanine green video angiography. The postoperative course was uneventful, and no ischemic lesions were confirmed on MR imaging.

**Conclusion:**

To the best of our knowledge, this is the first report of an ICA aneurysm with a DMCA and the AChA arising from the dome. In such cases, the anatomy of the DMCA and AChA should be well-characterized before treatment.

## Introduction

A duplicated middle cerebral artery (DMCA) is a normal variation of the middle cerebral artery (MCA), in which the MCA originates between the anterior choroidal artery (AChA) and the distal end of the internal carotid artery (ICA), and this passes into the sylvian fissure and perfuses part of the territory of the MCA (1, 2). The treatment of aneurysms at the origin of the DMCA has been reported previously (3–5). When treating aneurysms arising from the origin of the DMCA, it is important to preserve the AChA which branches nearby. We present a case in which clipping was performed for an ICA aneurysm with a DMCA and the AChA arising from the dome. There are no reports of aneurysms in which both a DMCA and the AChA branch from the dome. This report discusses the anatomical aspects of these aneurysms.

## Case Description

**Figure 1:**
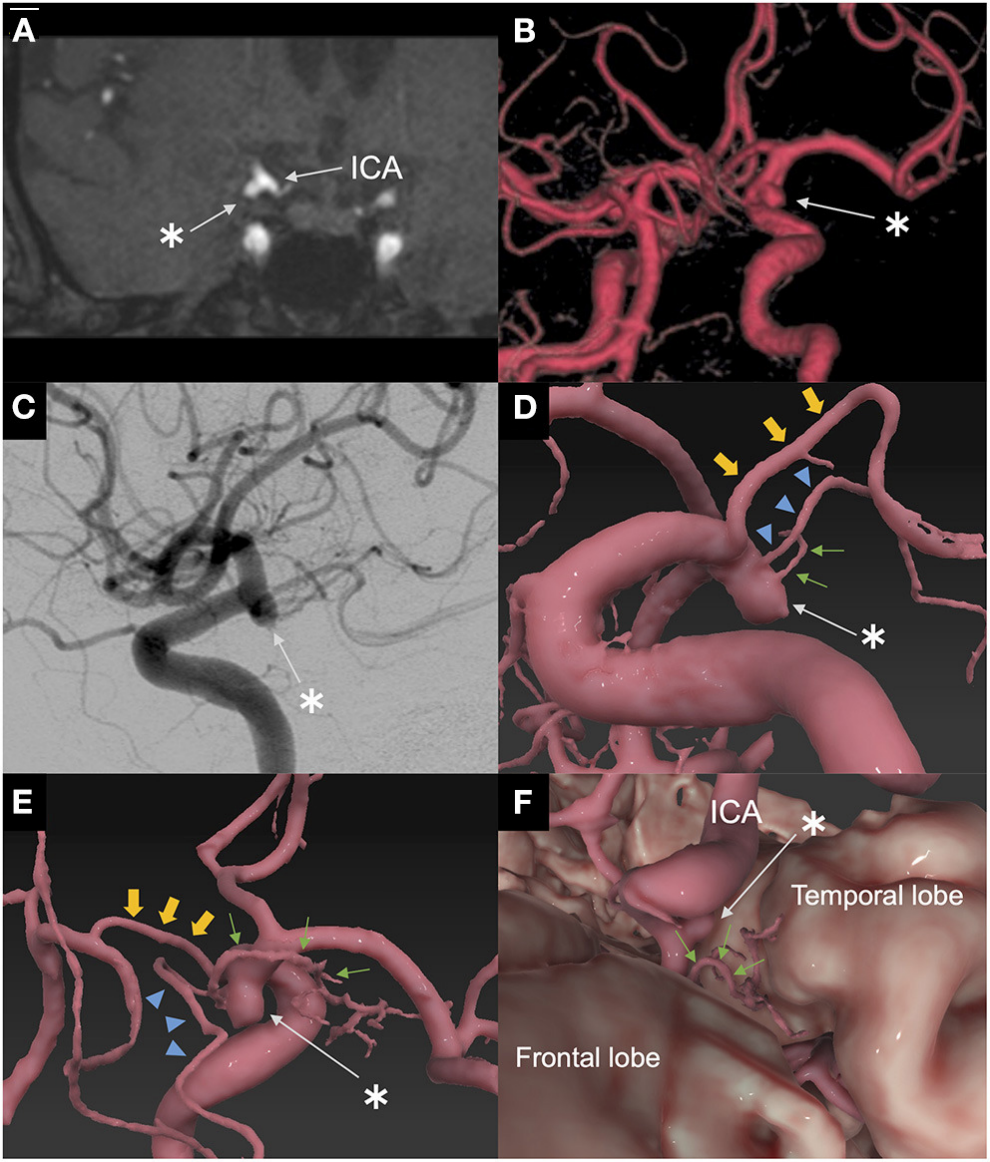
Preoperative imaging. **(A)** Coronal plane of time-of-flight magnetic resonance angiography on admission. Coronal plane of time-of-flight magnetic resonance angiography showed an unruptured aneurysm of the right internal carotid artery in contact with the temporal lobe. *: aneurysm. **(B)** Three-dimensional time-of-flight magnetic resonance angiography on admission. Three-dimensional time-of-flight magnetic resonance angiography showed an unruptured aneurysm of the right internal carotid artery supraclinoid portion. *: aneurysm. **(C)** Lateral view of digital subtraction angiography of the right internal carotid artery. Lateral view of digital subtraction angiography of the right internal carotid artery showing an aneurysm associated with a duplicated middle cerebral artery and anterior choroidal artery. *: aneurysm. **(D)** Fusion three-dimensional computer graphics integrating MR imaging/MR angiography and three-dimensional rotational angiography. Fusion three-dimensional computer graphics showed that the duplicated middle cerebral artery and the anterior choroidal artery originated from the dome. *: aneurysm; small arrow (green): duplicated middle cerebral artery; arrowhead (blue): anterior choroidal artery; large arrowhead (yellow): posterior communicating artery. **(E)** Fusion three-dimensional computer graphics. Fusion three-dimensional computer graphics shows that the duplicated middle cerebral artery passed through the sylvian fissure along the M1 segment of the MCA. *: aneurysm; small arrow (green): duplicated middle cerebral artery; arrowhead (blue): anterior choroidal artery; large arrowhead (yellow): posterior communicating artery. **(F)** Fusion three-dimensional computer graphics. Fusion three-dimensional computer graphics showed that the duplicated middle cerebral artery perfused the anterior temporal lobe. *: aneurysm; small arrow (green): duplicated middle cerebral artery. Fusion three-dimensional computer graphics were reconstructed by GRID 1.1 software (Kompath Inc., Tokyo, Japan).

**Figure 2:**
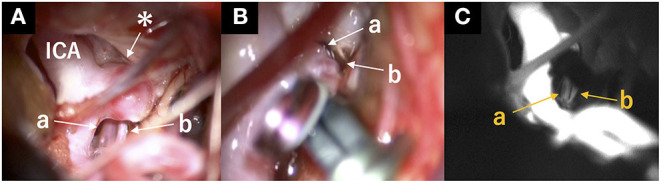
Intraoperative view. **(A)** Intraoperative view indicated the structures surrounding the aneurysm before clipping. *: aneurysm; a: anterior choroidal artery; b: duplicated middle cerebral artery. **(B)** Two titanium clips were combined and applied to occlude most part of the aneurysm, while confirming the patency of the DMCA and the AChA. a: Anterior choroidal artery; b: duplicated middle cerebral artery. **(C)** Patency of the duplicated middle cerebral and anterior choroidal arteries was confirmed using indocyanine green video angiography after clipping. a: anterior choroidal artery; b: duplicated middle cerebral artery.

A 64-year-old man was referred to our hospital because of an unruptured right ICA aneurysm that was detected incidentally on time-of-flight MR angiography (Figures 1A,B). Cerebral digital substruction angiography revealed an unruptured aneurysm with a maximum diameter of 4.3 mm at the supraclinoid portion of the right ICA (Figure 1C), and three-dimensional rotational angiography demonstrated that a DMCA and the AChA originated from the dome (Figure 1D). Additionally, fusion three-dimensional computer graphics integrating MR imaging and digital substruction angiography revealed that the DMCA passed through the sylvian fissure along the M1 segment of the MCA (Figure 1E) and perfused the anterior temporal lobe (Figure 1F). The fusion three-dimensional computer graphics was reconstructed using GRID 1.1 (Kompath Inc., Tokyo, Japan), utilizing the multi-threshold technique, as described previously (6–8). In summary, preoperative images were the output from the DICOM (digital imaging communication in medicine) format; they were imported into the image processing software GRID, which implements automatic registration of multiple imaging modalities. The multi-threshold is a method for extracting both thick and thin blood vessels with different threshold values. Microvessels can be visualized with less noise using this method (7). The patient had a family history of subarachnoid hemorrhage. The aneurysm increased to 4.3 mm after 10 years of follow up; it was 3 mm at the time of detection. Therefore, we determined that an intervention was required. The risk of occlusion of the arteries branching from the dome was estimated to be high if endovascular treatment was performed. In order to preserve the incorporated branch arteries, we decided to perform clipping. A right frontotemporal craniotomy was performed using a transsylvian approach to the aneurysm. Intraoperative findings showed that the DMCA and the AChA branched from the dome of the ICA aneurysm (Figure 2A). Two titanium clips were combined and applied to occlude most part of the aneurysm, while confirming the patency of the DMCA and the AChA (Figure 2B). Aneurysm obliteration and the patency of the parent and branch vessels were confirmed using intraoperative microvascular Doppler ultrasonography and indocyanine green video angiography (Figure 2C). Postoperatively, there were no neurological deficits or ischemic lesions on MR imaging.

## Discussion

To our knowledge, this is the first report of an ICA aneurysm with a DMCA and the AChA arising from the dome that was successfully treated without complications. Here, we discuss the anatomical aspects of this aneurysm and the importance of preserving the incorporated branch vessels.

The incidence of DMCA ranges from 0.7 to 2.9% (9, 10). The DMCA arises between the origin of the AChA and the distal end of the ICA. Both the AChA and MCA originate from the cranial division of the ICA, and the AChA is embryologically earlier than the MCA (11). Komiyama et al. (1) described the DMCA as an anomalous early ramification of the early branch of the MCA, which originates from the distal end of the ICA. DMCA is classified into two types, A and B, based on the point of origin. A Type A DMCA arises from the top of the ICA, while a Type B DMCA originates from the ICA between the AChA and proximal portion of the ICA bifurcation (12, 13). Most DMCA aneurysms are type B (5, 14). To the best of our knowledge, 42 cases of DMCA aneurysms have been reported (4, 5, 15–21). Most of these aneurysms are IC-DMCA aneurysms of Type B DMCA. There were five cases of rare variations among the DMCA aneurysms (Table 1) (15, 21–24), including three cases of aneurysms on DMCA. Theoretically, aneurysms that involve AChA, A1, or M1 could be considered as DMCA aneurysms; however, aneurysms that involve AChA, as in the present case, were not detected. Kai et al. (13) estimated that type B DMCA is exposed to higher hemodynamic stress because the angle between the ICA and type B DMCA is sharper than that of type A. Moreover, previous studies have reported that aneurysms associated with type B DMCA are at a high risk of rupture even if they are small in size. Therefore, aggressive treatment could be considered for such aneurysms, as in our case (13, 17, 25).

**Table 1:** Rare variations of DMCA aneurysms.

**Year**	**References**	**Age (years)**	**Sex**	**Size**	**Onset type**	**Case presentation**	**Treatment**
2004	Uchino et al. (22)	45	F	N/A	Ruptured	Saccular AN originated from DMCA trunk	Clipping
2010	Otani et al. (23)	66	F	5–10 mm	Ruptured	ICA AN at the origin of DMCA associated with accessory MCA and MCA aplasia	Clipping
2011	Takahashi et al. (24)	62	F	<5 mm	Ruptured	Kissing AN of ICA: ACHA was situated between two AN and DMCA originated from distal AN	Coiling
2012	LaBorde et al. (21)	34	M	10 mm	Unruptured	Fusiform AN originated from DMCA trunk	Trapping + STA-DMCA bypass
2018	Mori et al. (15)	62	M	<5 mm	Unruptured	Saccular AN originated from DMCA trunk	Observation

*AChA, anterior choroidal artery; AN, aneurysm; DMCA, duplicated middle cerebral artery; ICA, internal cerebral artery; MCA, middle cerebral artery; N/A, not assessed; STA, superficial temporal artery*.

The differential diagnosis of ICA aneurysm with DMCA and AChA arising from the dome included an aneurysm that involves the double AChA. According to Lasjaunias (26), the origin of the AChA is located posterolateral to the supracavernous portion of the ICA, between the posterior communicating artery and the ICA bifurcation. The AChA passes posterolaterally above the medial part of the uncus, along the optic tract, and laterally curves to reach the lateral geniculate body in the cisternal segment. Usually, the AChA gives off one or two branches that terminate at the medial wall of the temporal lobe. Double AChAs were found in 4–13% of cases (27–29), and their origins were classified into two types. One consists of two separate arteries arising from the ICA, and the other arises from the ICA as a single artery but immediately divides into two trunks. If there are double AChAs, the more distal branch terminates in the medial temporal lobe and the more proximal branch nourishes the remaining anterior choroidal field (29). Aneurysms involving a double AChA, which appear similar to the images of our case, have been reported (30, 31). Generally, the DMCA runs through the sylvian fissure and supplies the anterior and/or middle temporal territories (1). The present case is thought to be a DMCA because the artery originated between the AChA and the distal end of the ICA, passed through the sylvian fissure along the M1 segment of the MCA and perfused the anterior temporal lobe. Embryologically, the DMCA cannot originate proximal to the AChA because the AChA appears earlier in development. Uchino et al. (32) reported a case of DMCA arising from the origin of the AChA. In that case, the common origin of the DMCA and AChA was confirmed, and infundibular dilatation was indicated in the ICA-AChA-DMCA junction. It is assumed that the aneurysm in this case was the result of a DMCA aneurysm involving the AChA, an AChA aneurysm involving a DMCA, or an aneurysm occurring at the common origin of a DMCA and the AChA.

Miyoshi et al. reported a case of aphasia after clipping a DMCA aneurysm (20). DMCAs frequently involve perforating arteries (9, 33). In addition, a DMCA can potentially supply collateral blood flow to the MCA territory in cases of MCA occlusion (34). Thus, blood flow in the DMCA should be preserved. Furthermore, ischemia of the territory of the AChA causes severe neurological deficits (35, 36). Friedman et al. (37) showed that the AChA originates from the dome in 18% of AChA aneurysms, and the ischemic complication rate associated with treatment was even higher in such cases. As mentioned above, when treating aneurysms with a DMCA and the AChA originating from the dome, preserving these important branching vessels should be considered.

## Conclusion

To the best of our knowledge, this is the first report of an ICA aneurysm with a DMCA and the AChA arising from the dome. In such cases, the anatomy of the DMCA and AChA must be well-characterized before treatment is initiated.

## Data Availability Statement

The original contributions presented in the study are included in the article, further inquiries can be directed to the corresponding author.

## Ethics Statement

Written informed consent was obtained from the individual for the publication of any potentially identifiable images or data included in this article.

## Author Contributions

SM certifies that all authors have participated and have been involved in the cases presented and/or in the elaboration of the present manuscript. All authors contributed to the article and approved the submitted version.

## Conflict of Interest

The authors declare that the research was conducted in the absence of any commercial or financial relationships that could be construed as a potential conflict of interest.

## Publisher's Note

All claims expressed in this article are solely those of the authors and do not necessarily represent those of their affiliated organizations, or those of the publisher, the editors and the reviewers. Any product that may be evaluated in this article, or claim that may be made by its manufacturer, is not guaranteed or endorsed by the publisher.
